# Whole-liver transcatheter arterial chemoinfusion and bland embolization with fine-powder cisplatin and trisacryl gelatin microspheres for treating unresectable multiple hepatocellular carcinoma

**DOI:** 10.1007/s11604-020-01078-1

**Published:** 2021-01-02

**Authors:** Akihiro Imamura, Hidetoshi Taguchi, Hideyuki Takano, Hiroyuki Funatsu, Kazuyoshi Nakamura, Hidehito Arimitsu, Satoshi Chiba

**Affiliations:** 1 Division of Diagnostic Imaging, Chiba Cancer Center, 666-2, Nitonatyo, Chuouku, Chiba, 260-0801 Japan; 2 Department of Gastroenterology, Chiba Cancer Center, 666-2, Nitonatyo, Chuouku, Chiba, 260-0801 Japan; 3 Department of Hepato-Biliary-Pancreatic Surgery, Chiba Cancer Center, 666-2, Nitonatyo, Chuouku, Chiba, 260-0801 Japan

**Keywords:** Hepatocellular carcinoma, TACE, Fine-powder cisplatin, Trisacryl gelatin microsphere

## Abstract

**Purpose:**

To evaluate the safety and effectiveness of whole-liver transcatheter arterial chemoinfusion and bland embolization (TACBE) with fine-powder cisplatin and trisacryl gelatin microspheres for the treating unresectable multinodular hepatocellular carcinoma (HCC).

**Materials and methods:**

The medical records of all patients who underwent TACBE sessions were retrospectively reviewed. 15 patients (11 men, 4 women; mean age, 72.5 years) and 22 procedures (BCLC B;17 C;5) were included in the analysis. The cisplatin resulting solution and microspheres were infused through a microcatheter placed nonselectively. Overall survival (OS) was defined as the time from commencement of initial TACBE until any cause of death. Toxicity was assessed by the CTCAE version 5.0, and the tumor response was evaluated by the mRECIST. Liver function was assessed by the albumin–bilirubin (ALBI) score.

**Results:**

The 1-year OS rate was 64.6% (95% CI 0.438–0.955). Severe adverse effects were not observed except for grade 3 increase in the ALT, ALT, vasovagal episode. The objective response and disease control rare were 54.5% and 68.2%, respectively. The ALBI scores from pre-treatment to the follow-up ranged from − 2.39 to − 2.26 (*p* = 0.38).

**Conclusion:**

Whole-liver TABCE with fine-powder cisplatin and trisacryl gelatin microspheres was well tolerated and effective in patients with multinodular HCC.

## Introduction

Hepatocellular carcinoma (HCC) is the third leading cause of deaths related to cancer worldwide and an important health concern [1–3]. The Barcelona Clinic Liver Cancer (BCLC) staging system is the most widely used treatment algorithm worldwide [4].

The current standard treatment for unresectable intermediate-stage HCC (BCLC Stage B, Child–Pugh A/B, and large or multifocal tumor without extrahepatic spread) is transarterial chemoembolization (TACE). The benefits of TACE include improved treatment response and liver function [5]. However, in terms of tumor burden, BCLC stage B includes heterogeneous diseases. Patients beyond the up-to-seven criteria showed a significant deterioration in survival [6]. TACE is ineffective for substage B2 HCC, that means up-to-seven out. In such cases, TACE can further impair the hepatic functional reserve, resulting in poor prognosis [7].

DEB-TACE using drug-eluting bead, such as DC Bead™ (Biocompatibles International plc, Farnham, England) and Hepasphere™ (Biosphere Medical Rockland, MA, USA), are used worldwide. DEB-TACE using DC Bead was associated with improved tolerability [8]. However, these embolization materials are more expensive than other materials, such as Lipiodol, porous gelatin particles, and Embosphere.

Sorafenib treatment was shown to significantly prolong the overall survival (OS) in patients with advanced stage HCC [9, 10]. Lenvatinib is associated with better OS and better preservation of liver function than TACE in patients with HCC beyond the up-to-seven criteria with Child–Pugh A liver function [11]. However, Sorafenib and Lenvatinib treatment can lead to grade ≥ 3 treatment-emergent adverse events (TEAEs), such as hypertension, diarrhea, decreased appetite, decreased weight, and palmar–plantar erythrodysesthesia, and causes drug discontinuations. Thus, TACE may also be an option for patients with HCC beyond the up-to-seven criteria.

Whole-liver TACE for multinodular HCCs may cause severe liver damage and have fewer benefits for patients. Thus, to prevent liver failure, we performed whole-liver transcatheter arterial chemoinfusion and bland embolization (TACBE) with fine-powder cisplatin solution and trisacryl gelatin microspheres for multinodular HCC. This retrospective study aimed to evaluate the safety and efficacy of whole-liver TACBE with fine-powder cisplatin and trisacryl gelatin microspheres for treating unresectable multinodular HCC in our center.

## Materials and methods

This study was approved by the local ethics committee of our hospital (Approval number, R01-145).

All procedures performed in studies involving human participants were in accordance with the ethical standards of the institutional and/or national research committee and with the 1964 Helsinki declaration and its later amendments or comparable ethical standards.

Informed consent was obtained in the form of opt-out on the website of our hospital.

### Patients

We retrospectively analyzed the medical records of all patients who underwent TACBE procedures with fine-powder cisplatin solution and trisacryl gelatin microspheres between September 2014 and March 2019.

TACBE was performed for intermediate or Advanced Stage HCC Patients beyond up-to-seven criteria in dynamic CT, dynamic MRI, or CT during hepatic arteriography (CTHA) in our center. The criteria for TACBE were as follows: (1) unresectable hypervascular HCC confirmed radiologically in dynamic CT, dynamic MRI or CTHA; (2) tumor burden beyond up-to-seven criteria; (3) Child–Pugh class A or B liver function; and (4) Eastern Cooperative Oncology Group performance status 0, 1, or 2.

**Fig. 1:**
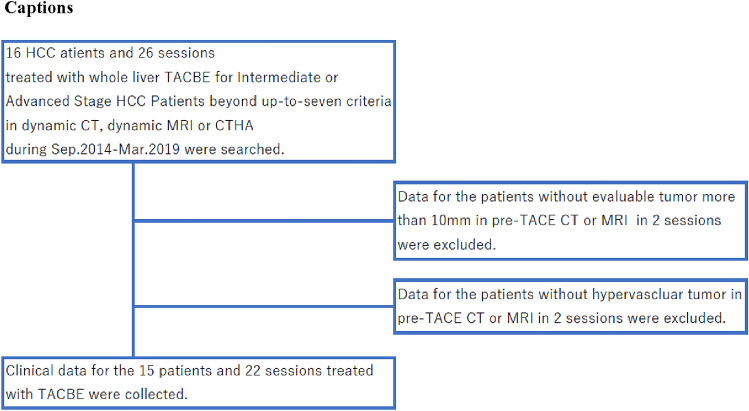
Inclusion and exclusion criteria used to select patients for this retrospective study from among 16 patients and 26 sessions with hepatocellular carcinoma (HCC) treated with whole liver TACBE

We performed TACBE 26 sessions for 16 patients. 2 patients had extrahepatic metastases, including metastases to the adrenal gland and lymph nodes. Therefore, we strongly recommended Sorafenib treatment. Despite our recommendations, they refused the treatment because of AEs and economic reasons, and expressed their desire to be treated with TACE. A total of 4 sessions were excluded from this study, because the patients had no evaluable tumors. 15 patients who underwent 22 TACBE procedures were included in this study (Fig. 1). Patients’ characteristics and disease characteristics in each session are summarized in Tables 1 and 2.

**Table 1:** Patients’ characteristics

Age	
Mean	72.5
Range	38–85
Sex	
Male	11
Female	4
ECOG performance status	
0	12
1	2
2	1
Etiology	
HCV	12
HBV	0
HBV and HCV	0
Other	3
Previous therapy	
None	2
TACE	5
Lobectomy	1
Lobectomy + TACE	7
HCC status	
New	2
Recurrent	13

**Table 2:** Disease characteristics in each session. 1 patient performed 2 sessions had lymph node metastasis

No. of tumors	
~ 5	2
5–10	10
11–20	8
> 20	2
Maximum tumor diameter (mm)	
Mean	27.6
Range	13–50
Sum of measurable targeted lesions (mm)	
Mean	77.8
Range	26–150
BCLC staging	
B	17
C	5
Child–Pugh class	
A	19
B	3
Portal invasion factor	
0	21
1	1

2 patients performed 1 session and 2 sessions, respectively, had adrenal metastasis

For evaluating hepatic arterial injury (HAI), a total of 8 sessions were excluded from this study, because the patients were not performed TACE sessions after TACBE. 8 patients who underwent 14 TACBE procedures (5 patients, 3 patients and 1 patient were performed 1 session, 2 sessions, and 3 sessions, respectively) were included in this study.

**Table 3:** Adverse events

	CTCAE version 5 gade					
	All grades		3		4	
	No. of events	(%)	No. of events	(%)	No. of events	(%)
Hyperbilirubinemia	6	27.3	0	0	0	0
Elevated AST	22	100	7	31.8	0	0
Elevated ALT	21	95.5	3	13.6	0	0
Elevated creatinine	8	36.4	0	0	0	0
Anorexia	13	59.1	0	0	0	0
Nausea	4	18.2	0	0	0	0
Fatigue	8	36.4	0	0	0	0
Fever without neutropenia	8	36.4	0	0	0	0
Vomiting	3	13.6	0	0	0	0
Abdominal pain	6	27.3	0	0	0	0
Hypertension	0	0	0	0	0	0
Suspected liver abscess	0	0	0	0	0	0
Vasovagal episode	1	4.5	1	4.5	0	0
Hypotension	1	4.5	0	0	0	0
Hiccups	1	4.5	0	0	0	0
Constipation	0	0	0	0	0	0

### TACBE technique

To prepare the cisplatin solution, we added 70 mL of saline solution warmed to 50 °C to a vial containing 100 mg of fine-powder cisplatin (IA Call; Nippon Kayaku). The solution containing 65 mg/mm^2^ of cisplatin was manually infused for 20 min through a microcatheter that was nonselectively placed in the proper hepatic artery to enable complete exposure of all tumors to the drug. If necessary, the drug was separately injected from the right or left hepatic artery to account for anatomical variations.

Following cisplatin infusion, all hepatic arteries were embolized with 100–300 μm trisacryl gelatin microspheres (Embosphere; Nippon Kayaku). However, microsphere injections into the cystic, left gastric, and right gastric arteries were avoided. Completion of the therapy was defined as the disappearance of all tumor enhancements on postembolization digital subtraction angiography of the proper hepatic artery. In cases of vascular lakes and arteriovenous shunts, porous gelatin particles (Gelpart; Nippon Kayaku) were used. Lipiodol (Guerbet, France) was not used. To prevent renal damage, 1000 and 1500 mL of electrolytes were administered over a period of 4 h before and 6 h after the procedure, respectively. Antiemetic agents, including a 5-HT3 antagonist and steroids, were prophylactically administered to reduce nausea and vomiting.

### Safety evaluation

The incidence and severity of AEs were assessed for all TACBE sessions. AEs were graded according to the Common Toxicity Criteria for Adverse Events (version 5). AEs occurring within 2 weeks after TACBE were considered treatment related, and whenever they occurred after 2 weeks, they were reported only if a causal correlation was suspected.

Liver function was assessed by the albumin–bilirubin (ALBI) score. The ALBI scores before TACBE, after TACBE, and during the follow-up period were compared. OS was defined as the time from initial TACBE with this method until any cause of death.

Hepatic arterial injury (HAI) caused by TACBE was assessed by the angiographic findings and CTHA imaging. We compared pre-TACBE with next TACE session. The criteria for the next TACE, including TACBE, conventional TACE (cTACE), and DEB-TACE, were as follows: (1) unresectable hypervascular HCC confirmed radiologically in dynamic CT or dynamic MRI; (2) Child–Pugh class A or B liver function; and (3) Eastern Cooperative Oncology Group performance status 0, 1, or 2. Two interventional radiologists (with 15- and 11-year clinical experience, respectively) assessed and a consensus interpretation was made. HAI was evaluated at each segmental hepatic artery using a three-grade scale: 1 = slight wall irregularity; 2 = overt stenosis; and 3 = occlusion. Distal hepatic arteries directly supplying a tumor were not evaluated, because the configuration of the arteries could be changed without HAI as a tumor shrinks or enlarges and could thus be confused with true HAI. The four, segmental, hepatic arteries were evaluated, that is, the right anterior segmental hepatic artery, right posterior segmental hepatic artery, left lateral segmental artery, and the left medial segmental hepatic artery.

### Efficacy evaluation

Overall survival (OS) was defined as the time from commencement of initial TACBE until any cause of death.

Tumor response was evaluated based on contrast-enhanced computed tomography (CT) findings of 20 sessions. In the remaining two sessions, tumor response was evaluated based on contrast-enhanced magnetic resonance imaging findings before TACBE and contrast-enhanced CT findings after TACBE. Tumor response rates were calculated for all treated patients. The best overall response was categorized according to the modified Response Evaluation Criteria in Solid Tumors (mRECIST).

### Statistical analysis

Data are expressed as the mean and standard deviation or median and range. Statistical analyses were performed using the Kaplan–Meier method and *t* test. A *p* value of < 0.05 was considered statistically significant. All analyses were performed using the “R”, version 3.6.1.

## Results

### Safety

Adverse events are summarized in Table 2. A transient, grade 3 increase in the levels of aspartate aminotransferase (AST) and alanine aminotransferase (ALT) was observed in seven (31.8%) and three (13.6%) sessions, respectively. A grade 3 vasovagal episode occurred in one session (4.5%). Grade 3 thrombocytopenia was observed in one session (4.5%), but platelet transfusion was not required. In that case, thrombocytopenia was observed before the TACBE period; therefore, this AE was likely not attributable to the study drug (Table 3).

**Fig. 2:**
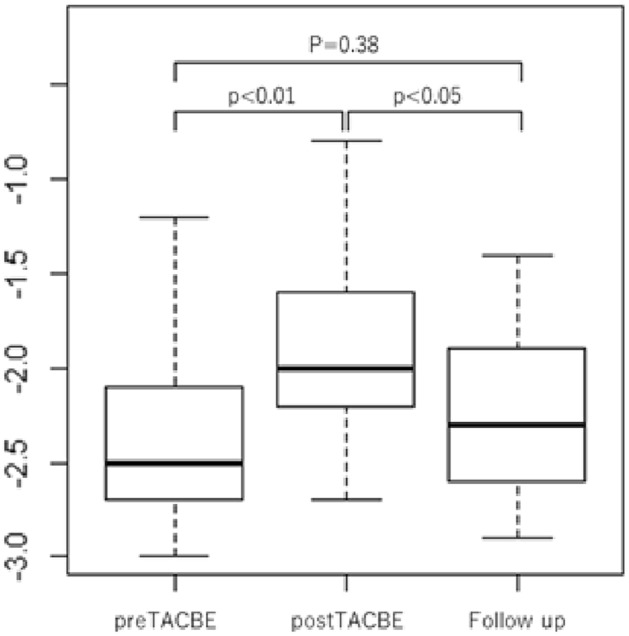
Albumin–bilirubin (ALBI) score over time. ALBI score was significantly worsened at post-TACBE (− 1.93) as compared with that at pre-TACBE (− 2.39) (*p* < 0.01). In contrast, ALBI score was maintained at the pre-TACBE (− 2.39) and at the follow-up (− 2.26) (*p* = 0.38)

The ALBI score before and after TACE and at pre-treatment and follow-up ranged from − 2.39 to − 1.93 (*p* < 0.01) and from − 2.39 to − 2.26 (*p* = 0.38) for 22 procedures, respectively. The mean date from TACBE to follow-up was 53.3 (range 19–126) days (Fig. 2).

**Fig. 3:**
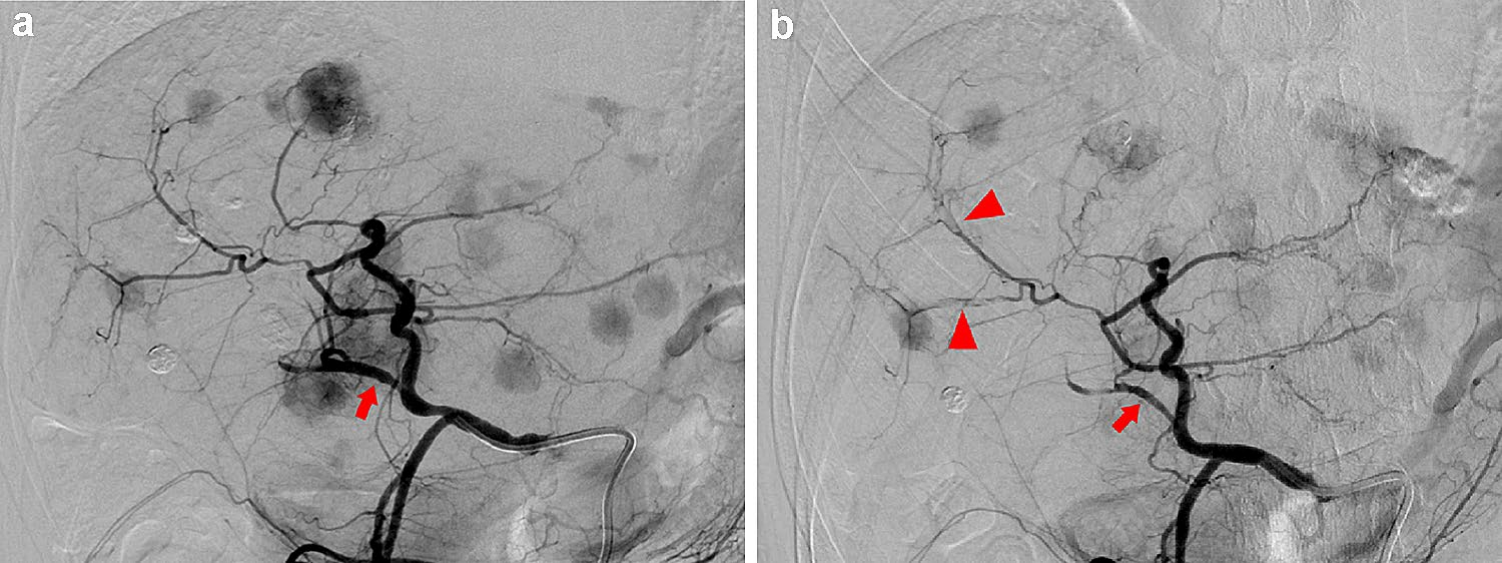
**a** Before 1st TACBE procedure for patient with the past of right lobectomy, the middle left hepatic artery was normal (arrow). **b** In the images obtained 98 days after 1st TACBE procedure, the middle left hepatic artery got narrower (arrow). Arterio-portal shunts were observed in segment 4 (arrowhead)

HAI was observed in 0 during 14 sessions of TACBE. In one session, the left medial segmental hepatic artery got narrower, but we assessed that was caused by tumor shrinks (Fig. 3a, b).

### Efficacy

**Fig. 4:**
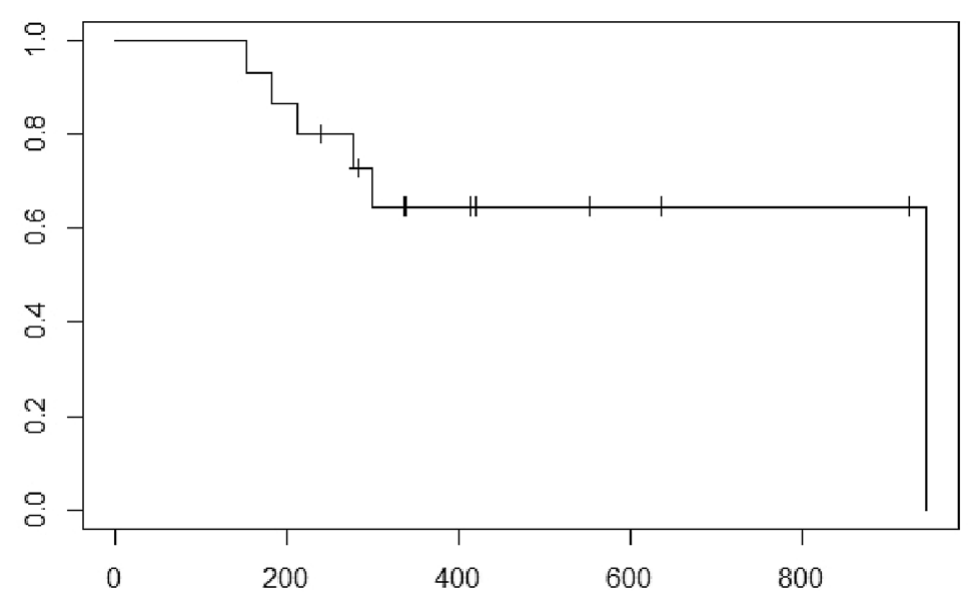
The Kaplan–Meier curve shows the OS after the first TACBE protocol therapy. The 1-year survival rates were 64.6% (95% CI 0.438–0.955). The median survival was not reached

The mean follow-up period was 426.1 (range 151–980) days. The 1-year OS rate was 64.6% (95% CI 0.438–0.955; Fig. 4). The median survival was not reached. 6 patients (40%) died as a result of cancer progression. 1 patient was transferred to the hospital which was near to the patient’s house for treatment on the patient hope. 1 patient was transferred to a hospital for treatment with a molecular targeted agent. 2 patients were transferred to a hospital for palliative care.

**Fig. 5:**
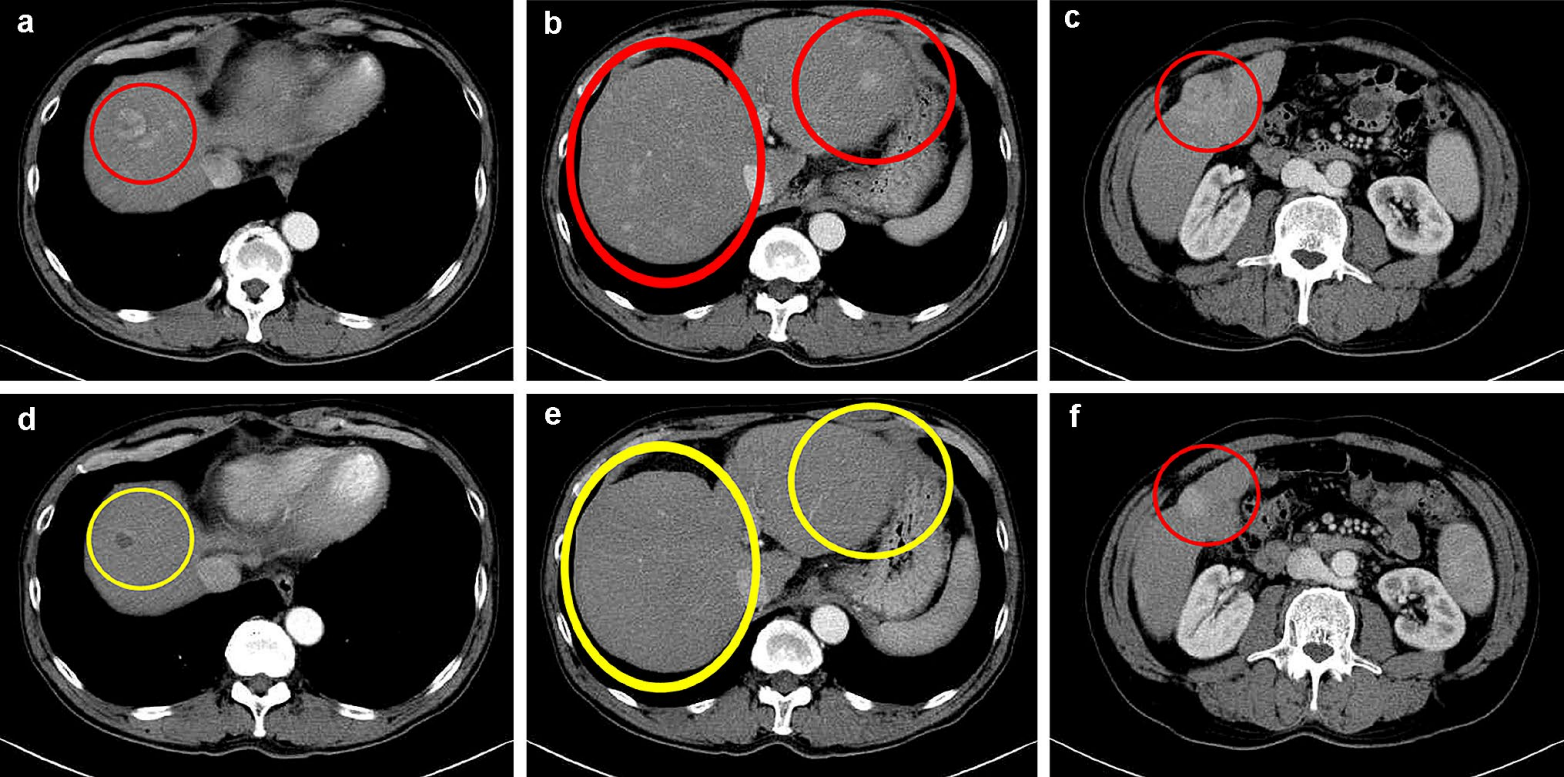
**a**–**c** Before 1st TACBE procedure, there were a large number of tumors in CECT image. **d**–**f** In the images obtained 36 days after 1st TACBE procedure, majority of tumors disappeared. The tumor in segment 8 exhibited low-density mass reflecting necrosis, and the tumor in segment 5 appeared smaller in size. This patient was determined to have achieved partial response

Complete response, partial response, stable disease, and progressive disease were observed in 0, 12 [54.5% (95% CI 0.439–0.652)], 3 [13.6% (95% CI 0.063–0.210)], and 7 sessions [31.8% (95% CI 0.219–0.417)], respectively. According to mRECIST, the objective response and disease control rates were 54.5% (95% CI 0.439–0.652) and 68.2% (95% CI 0.583–0.781), respectively. In all PD sessions, the sum of the longest diameters of target lesions decreased, but new lesions appeared. The mean date from TACBE to follow-up CT was 86.7(range 29–166) days. CT images of a patient are showed in Fig. 5a–f.

## Discussion

In this study, TACBE was well tolerated and effective in patients with multifocal HCC beyond up-to-seven criteria.

To satisfy the requirements of sparing liver function and treating whole liver lesions in one session, we performed whole liver TACBE for treating multifocal HCC. Whole-liver TACE with Lipiodol and porous gelatin particles causes severe adverse events. For preventing severe liver damage, bi-lobar multiple HCC are sometimes treated with TACE in two processes within short term. However, while waiting for next treatment for alternative lobe, tumors can become larger. DEB-TACE is known that the adverse effects are lower than ctace, though whole liver DEB-TACE may also cause adverse effects and hepatic failure. Bland embolization is tolerated; however, it is not recommended.

DEB-TACE using DC Bead was associated with improved tolerability, such as abdominal pain [8, 12]. Conversely, in a preclinical study with young Yucatan pigs, DEB-TACE with DC beads loaded with doxorubicin resulted in pan-necrosis of the adjacent hepatic tissue, notable amounts of acute neutrophilic inflammation, moderate amounts of portal fibrosis, moderate biliary hyperplasia, and mild arterial and venous hyperplasia. In that study, authors also reported that bland embolization resulted in mostly non-necrotic vascular changes, particularly vasculopathy, with no hepatic necrosis and with occasional biliary hyperplasia and portal fibrosis [13]. Three key conclusions were drawn from this study: (1) in the presence of doxorubicin, DEB-TACE caused hepatic tissue damage, (2) repeated DEB-TACE sessions could cause hepatic failure, and (3) bland embolization was more tolerable for normal liver tissue than DEB-TACE.

Bland embolization using only microspheres without loading anticancer drugs was reported. Bland embolization showed that the median survival time was 21 months, with 1-, 2-, and 3-year OS rates of 66%, 46%, and 33%, respectively [14]. However, a prospective randomized comparison of chemoembolization with doxorubicin DEB and bland embolization for hepatocellular carcinoma showed that DEB-TACE resulted in a better local response, fewer recurrences, and a longer time to progression than bland embolization [15]. Nevertheless, the European Association for the Study of the Liver (EASL) Clinical Practice Guidelines 2018 states that there is insufficient evidence to recommend bland embolization [16]. Therefore, treating with embolization particle and anticancer drug are recommended.

There is hesitation among clinicians for performing whole-liver TACE for bi-lobar multiple HCC in one session because of AEs. In the PRECISION V study, for patients with bi-lobar disease who could not be treated super selectively in a single treatment, a second embolization of the alternative lobe was performed within 3 weeks of the first procedure if there was no contraindication owing to systemic toxicity and/or clinical performance [8].

The Japan Interventional Radiology in Oncology Study Group (JIVROSG, study code 0401) reported on whole-liver TACE using fine-powder cisplatin solution and porous gelatin particles. In this phase II study, nonselective TACE was performed with 65 mg/m^2^ of cisplatin solution and porous gelatin particles. Hyperbilirubinemia, elevated AST level, elevated ALT level, and grade 3 nausea were observed in 2.2%, 28.3%, 21.7%, and 2.2%, respectively. The tumor response rate was 65.2%. Thus, the authors concluded that the method was well tolerated and effective in patients with multifocal HCC [17]. In comparison, our method was associated with a lower incidence of AE, suggesting that our approach was also well tolerated.

One of the key points of HCC treatment is to preserve liver function as much as possible. Liver function is commonly evaluated using the Child–Pugh classification. Conversely, the ALBI score, which was recently developed, was calculated based on albumin and bilirubin levels. The ALBI grade is thought to be more accurate than the Child–Pugh classification score for assessing hepatic functional reserve [18].

A retrospective study comparing Lenvatinib and cTACE in patients with intermediate-stage hepatocellular carcinoma beyond the up-to-seven criteria showed that Lenvatinib was associated with better OS than TACE because of high objective response rate/clinical benefit rate/disease control rate, better progression-free survival, and better preservation of liver function. The changes in the ALBI scores from the baseline to the end of treatment were from − 2.61 to − 2.61 in the Lenvatinib group (*p* = 0.254) and from − 2.66 to − 2.09 in the cTACE group (*p* < 0.01), respectively [19]. ALBI grade 1 predicted a higher response rate, lower treatment discontinuation owing to adverse events and better outcomes with Lenvatinib treatment. The authors concluded that Lenvatinib treatment should be started at an early phase when liver function is preserved instead of repeating ineffective and/or liver function-impairing procedures, such as unselective TACE [20]. However, treatments with Lenvatinib and Sorafenib can cause grade ≥ 3 TEAEs, such as hypertension, diarrhea, decreased appetite, decreased weight, and palmar–plantar erythrodysesthesia. The reported drug discontinuations rate owing to Lenvatinib-related TEAEs is 9% [21]. These TEAEs generally lead to drug discontinuations and poor patient adherence to treatment. Therefore, TACE is also an option for HCC beyond up-to-seven criteria. By our method, the ALBI scores from pre-treatment to the follow-up period were from − 2.39 to − 2.26 (*p* = 0.38), showing that TACBE resulted in less liver damage than cTACE.

When selecting a treatment for patients with HCC beyond the up-to-seven criteria, invisible pre-cancerous lesions and multi-step changes of intramodular blood supply during hepatocarcinogenesis should be considered. The intranodular blood supply changed in accordance with hepatocarcinogenesis from a dysplastic nodule to overt HCC. The intranodular arterial supply is initially decreased during the early stages of hepatocarcinogenesis and is then increased in parallel with the increasing grade of malignancy of the nodules [22]. Patients with HCC beyond the up-to-seven criteria may have such an early HCC affecting the whole liver. Porous gelatin particles and microspheres cannot reach such tumors because of the narrow arteries. Selectively injecting lipiodol to the whole liver can reach tumors with narrow arteries, but it causes severe liver damage. By providing systemic therapy, such as administration of Lenvatinib and Sorafenib, along with chemoinfusion, such tumors can be reached. Thus, our method may be considered as beneficial for patients beyond the up-to-seven criteria.

Repeated TACE can cause HAI. HAI caused by TACE with cisplatin and Gelfoam was reported. In three or more sessions of TACE, the incidence of HAI was 24%. Increasing TACE causes increased incidence of HAI [23]. Although HAI was not observed in our study, the number of sessions is small. Repeated TACBE may cause HAI.

DC Bead and Hepasphere are also commercially available drug-eluting Microspheres in Japan. DC Bead and Hepasphere cost 99,000 yen in the Japanese insurance regime. Thus, repeated DEB-TACE causes high cost. On the other hand, Embosphere costs 26,500 yen in the Japanese insurance regime. Therefore, the cost of TACBE is lower than DEB-TACE.

The advantages of the method presented herein may be many and include ease of performance, low incidence of AEs, low risk of liver damage, no need for drug loading to the microspheres, low cost compared with DEB-TACE, and good overall response. This method may be an option for treating patients with bi-lobar multinodular HCC, those with multinodular HCC in the remnant liver after surgery, those classified as Child–Pugh B, those who discontinued drugs owing to TEAEs, or those refusing Sorafenib or Lenvatinib treatment.

## Limitations

Our study had several limitations. First, this was a retrospective study. The number of study participants was small. Furthermore, we did not compare this method with DEB-TACE, Lenvatinib, or Sorafenib treatment. Finally, patients in this study underwent a short-term evaluation.

## Conclusions

Whole-liver TACBE with fine-powder cisplatin and trisacryl gelatin microspheres was well tolerated and effective in patients with unresectable multinodular HCC. This method may be an option for patients with bi-lobar multinodular HCC, patients with multinodular HCC in the remnant liver after surgery, patients with Child–Pugh B HCC, those who discontinued drugs because of TEAEs, or those refusing Sorafenib or Lenvatinib treatments.
